# Innovative Design of Aerostatic Bearings with Enhanced Dynamic Stability Inspired by the Laval Nozzle Principle

**DOI:** 10.1007/s41871-025-00282-9

**Published:** 2026-01-19

**Authors:** Xiuyuan Chen, Xichun Luo, Wenkun Xie, Yankang Tian, Song Yang

**Affiliations:** 1 Centre for Precision Manufacturing, DMEM, University of Strathclyde, Glasgow, UK; 2 Innova Nanojet Ltd, Glasgow, UK

**Keywords:** Aerostatic bearing, Laval nozzle principle, Computational fluid dynamics simulation, Dynamic stability

## Abstract

Microvibrations caused by airflow self-excitation within pressurized air films significantly degrade the dynamic stability of aerostatic bearings. However, effectively controlling supersonic flow velocity, which is critical for suppressing the turbulent airflows that cause this self-excitation, remains a significant challenge in the current designs of aerostatic bearings. To address this gap, a novel aerostatic restrictor inspired by the Laval nozzle principle is proposed to enhance the dynamic stability of bearings by decelerating supersonic pressurized airflows. Computational fluid dynamics (CFD) simulations are conducted to elucidate the underlying mechanism by which the proposed restrictor improves performance (i.e., by suppressing turbulent airflows by mitigating adverse pressure gradients). On the basis of the CFD simulation results, the key geometrical parameters of the newly designed restrictor are identified. The effectiveness of the proposed restrictor is evaluated through experimental testing, with the results indicating that it achieves improved dynamic stability and reduced vibration amplitude compared with a conventional aerostatic restrictor design. This work is expected to advance the theory of restrictor design by enhancing the dynamic stability of aerostatic bearings.

## Highlights

1. Proposed a novel aerostatic restrictor design inspired by rocket-engine Laval nozzles.
2. Mitigated adverse pressure gradients by decelerating supersonic turbulent flow.
3. Identified suitable structural parameters to suppress negative airflow divergence and velocity concentration.

## Introduction

Aerostatic bearings have been widely used in ultraprecision equipment, such as machine tools, metrology devices, and cutting-edge lithography systems, given their low friction, high accuracy, and long service life [1, 2]. However, due to their inherently low damping characteristics, improving dynamic stability remains a significant research challenge for aerostatic bearing design.

Self-excited vibration is regarded as a major cause of dynamic instability of aerostatic bearings, especially in the absence of external disturbances. Insufficient bearing damping fails to quickly attenuate this vibration energy, causing it to persist during operations [3]. One severe consequence is the “pneumatic hammer,” characterized by large amplitudes and continuous whistling, which leads to periodic self-movement that may damage the bearing system [4]. This phenomenon can be avoided by optimizing restrictor selection, regulating air volume, and avoiding resonance [5, 6]. However, microvibrations caused by turbulent flow fluctuation are difficult to eliminate because of their low amplitudes and high randomness. This airflow turbulence is induced by the adverse pressure gradient created by shock waves. In aerostatic bearing films, shock waves arise when pressurized airflow transitions from supersonic to subsonic velocities. Their intensity is closely linked to the development of sonic regions, which depend on the local supersonic velocity around the aerostatic bearing feedhole [7, 8]. However, decreasing this supersonic velocity is challenging. Thus, inspired by the Laval nozzle concept used in rocket-engine design, a new aerostatic restrictor is proposed to decelerate this supersonic airflow by alleviating the adverse pressure gradient to enhance bearing dynamic stability.

Microvibrations, despite occurring at nanometer scales, can downgrade the accuracy of ultraprecision facilities equipped with aerostatic bearings [9]. Thus, minimizing or even eliminating such vibrations is a crucial task in aerostatic bearing design. One contributing factor is the turbulent airflow within the bearing film, where vortices interact dynamically and transfer energy across scales, resulting in pressure fluctuation [10]. Understanding this unstable pressurized airflow, particularly its pressure and velocity distributions, facilitates the bearing design process targeted at suppressing microvibrations [7]. To improve bearing dynamic instability, pressurized airflow dynamics are commonly regulated using innovative restrictor designs [11, 12]. By breaking large vortices into smaller ones, the arrayed microhole restrictor proposed by Chen et al. [13] suppressed the turbulent flow of the aerostatic bearing. To change the unstable airflow characteristics near the supply orifice, Li et al. [14] introduced a flow field disturbance structure into a pocketed restrictor to improve the microvibration of an aerostatic thrust bearing. To alter the transition of the airflow velocity direction within the recess, Feng et al. [15] designed three types of orifice restrictors with different air supply channel structures to suppress air vortex formation and thus improve bearing dynamic stability. However, current research has mainly focused on redesigning restrictors of the orifice or pocket aerostatic bearings to improve dynamic stability [16, 17]. To our best knowledge, the underlying mechanism of microvibration suppression using modified compound restrictors is seldom tackled.

Compound restrictors, which combine inlet and outlet restrictors, have attracted great attention because of their simplicity and cost-effective manufacturing, as well as their relatively good stiffness and load capacity [18, 19]. The outlet groove structure inside this kind of restrictor is crucial for regulating the pressure distribution within the air film, which determines the bearing static and dynamic performances [20]. However, it is still very challenging to modify this structure as the pressure distribution will be easily changed under the improper airflow hinder effect [21]. Thus, a newly designed, narrowed divergent groove outlet structure is integrated into the aerostatic restrictor design without disturbing the airflow.

This paper aims to design and create a novel diverging groove structure to improve the dynamic stability of aerostatic bearings by incorporating the Laval nozzle principle into the outlet restriction design. The underlying mechanism and operational feasibility of the Laval-nozzle-inspired aerostatic restrictor application to suppressing microvibrations are revealed through computational fluid dynamics (CFD) simulations and verification experiments.

The design principle of the proposed restrictor is presented in Sect. 2. The preliminary design and CFD modeling setup are described in Sect. 3. Section 4 discusses the influencing mechanism of different restrictor key structural parameters on pressurized flow status to identify the bearing configuration. In Sect. 5, a set of dynamic stability testing experiments is performed on a prototype of an aerostatic bearing stage to verify the effectiveness of the new aerostatic restrictor. Section 6 presents the conclusions.

## Methodology

**Fig. 1:**
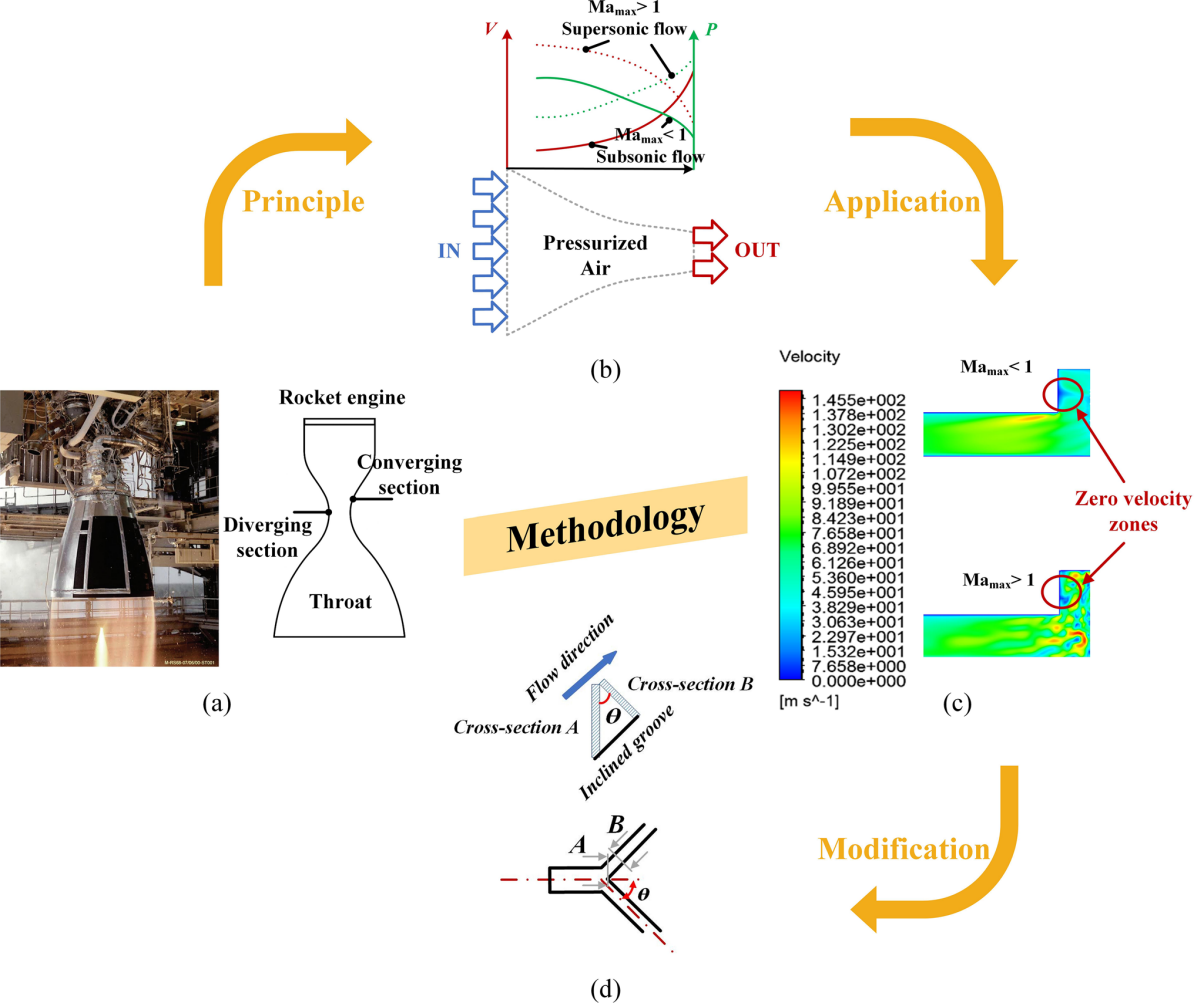
Introduction of the Laval nozzle principle into aerostatic bearing restrictor design: **a** rocket engine nozzle; **b** Laval nozzle principle; **c** simulation results for the narrow groove in subsonic and supersonic velocity conditions; **d** schematic of the proposed diverging groove structure

As shown in Fig. 1a, a Laval nozzle is a rapidly converging and gradually diverging tubular structure regularly used to accelerate compressible fluids to supersonic speeds, particularly in rocket-engine nozzle applications [22]. Inspired by this principle, we explored its inverse application to improve the dynamic stability of bearings. Specifically, a reverse design approach with the deceleration of supersonic airflow was utilized. As demonstrated in Fig. 1b, the velocity (*V*) of the pressurized supersonic airflow decreased upon entering the narrowed bearing outlet groove because of the adverse pressure (*P*) gradient. Here, the Laval nozzle principle was applied inversely, with the outlet groove decelerating supersonic airflow by mitigating adverse pressure gradients. This configuration suppressed vortex formation and turbulence, thereby improving the dynamic stability of the aerostatic bearing. However, narrowing this groove structure without disturbing air flowability remains a research challenge.

To better understand this phenomenon, the maximum Mach number (Ma_max_) was analyzed, as shown in Fig. 1c, reflecting the development degree of the sonic region [8]. Then, CFD simulations showed the velocity distributions of the narrowing groove by half directly under subsonic (Ma_max_ < 1) and supersonic (Ma_max_ > 1) conditions. In both cases, the absolute supply pressure (*P*_s_) was set to 0.5 MPa, whereas the film thicknesses (*h*_*ij*_) were set to 8 and 20 μm, respectively. When Ma_max_ < 1, no evident unstable airflow was observed. In contrast, when Ma_max_ was over 1, clearly unstable airflow was observed, while the maximum velocity of the narrow groove was decreased to 170.86 m/s from 209.62 m/s. This demonstrated the effectiveness of applying the Laval nozzle design principle. However, this simple groove structure obviously hindered pressurized air flowability, as supported by the occurrence of zero velocity zones.

To overcome this limitation, a novel diverging groove structure was proposed to maintain smooth pressurized airflow, as shown in Fig. 1d. Taking 45° as the diverging angle, the area ratio of cross-section *A* to cross-section *B* was equal to $\sqrt{2}$ along the inclined groove, demonstrating the narrowing of the groove width. This bearing restrictor design concept not only mitigates flow resistance but also improves the dynamic stability of aerostatic bearings. By directly linking the outlet restrictor’s geometry with airflow regulation, the Laval nozzle principle provides a theoretical foundation for the diverging groove structure applied in this study.

## Design and Modeling of Bearing Outlet Restriction

### Preliminary Design

**Fig. 2:**
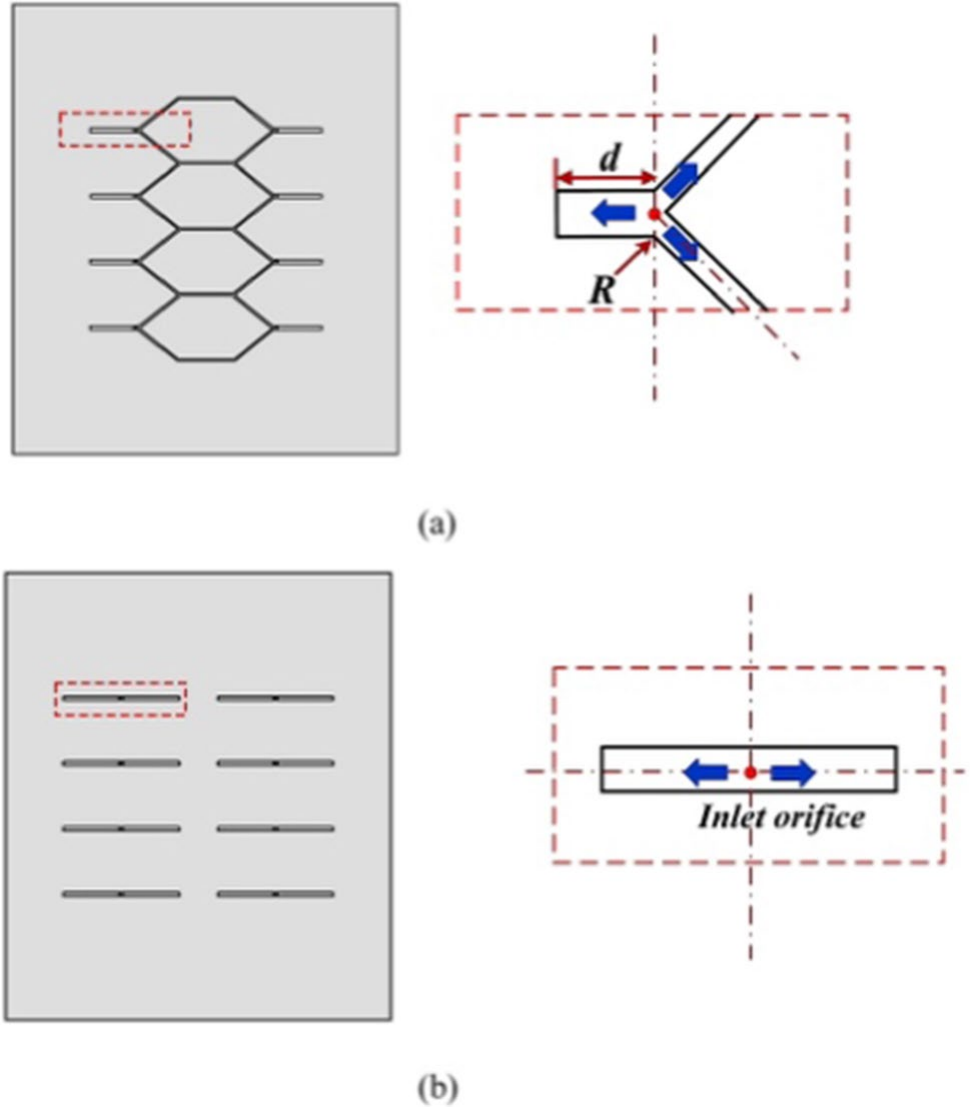
Key geometrical parameters of compound **a** diverging and **b** linear groove aerostatic bearings

Figure 2a and b show the schematic for the designed diverging and linear groove structures on the aerostatic bearing, respectively. The orifice distance *(d*) and round radius (*R*) in Fig. 2a were set to vary in the range of 4.5–4.8 mm and 0.2–0.5 mm, respectively, to evaluate their influence on airflow stability. On this basis, the operating performances of the aerostatic bearing under varying *h*_*ij*_ and *P*_s_ could be analyzed, which contributed to the final verification of key structural parameters (i.e., *d* and *R*). The ranges of *h*_*ij*_ and *P*_s_ were adopted from previous works [1, 23].

The bearing width and length were 40 and 58 mm. Meanwhile, the orifice diameter (*d*_o_), groove depth (*H*_g_), groove width (*G*_w1_), diverging groove width (*G*_w2_), and diverging angle (*θ*) were set to 0.4 mm, 15 µm, 0.4 mm, 0.2 mm, and 45°, respectively. The 45° angle was chosen as a representative case to provide a practical balance between the expansion ratio and structural constraints while illustrating the feasibility of applying the Laval nozzle principle to suppress turbulence.

### Design Workflow

In aerostatic bearings, the local supersonic flow region around the supply orifice influences the development of turbulent flow [24]. To ensure that the proposed diverging groove structure can properly restrict turbulent flow, accurately positioning it relative to the inlet restriction is crucial. Thus, *d* must be determined. Moreover, the existence of an improper transition corner potentially causes velocity concentration, which may also influence the airflow status within the inclined groove. Thus, *R* is another critical factor in terms of the outlet restriction design.

**Fig. 3:**
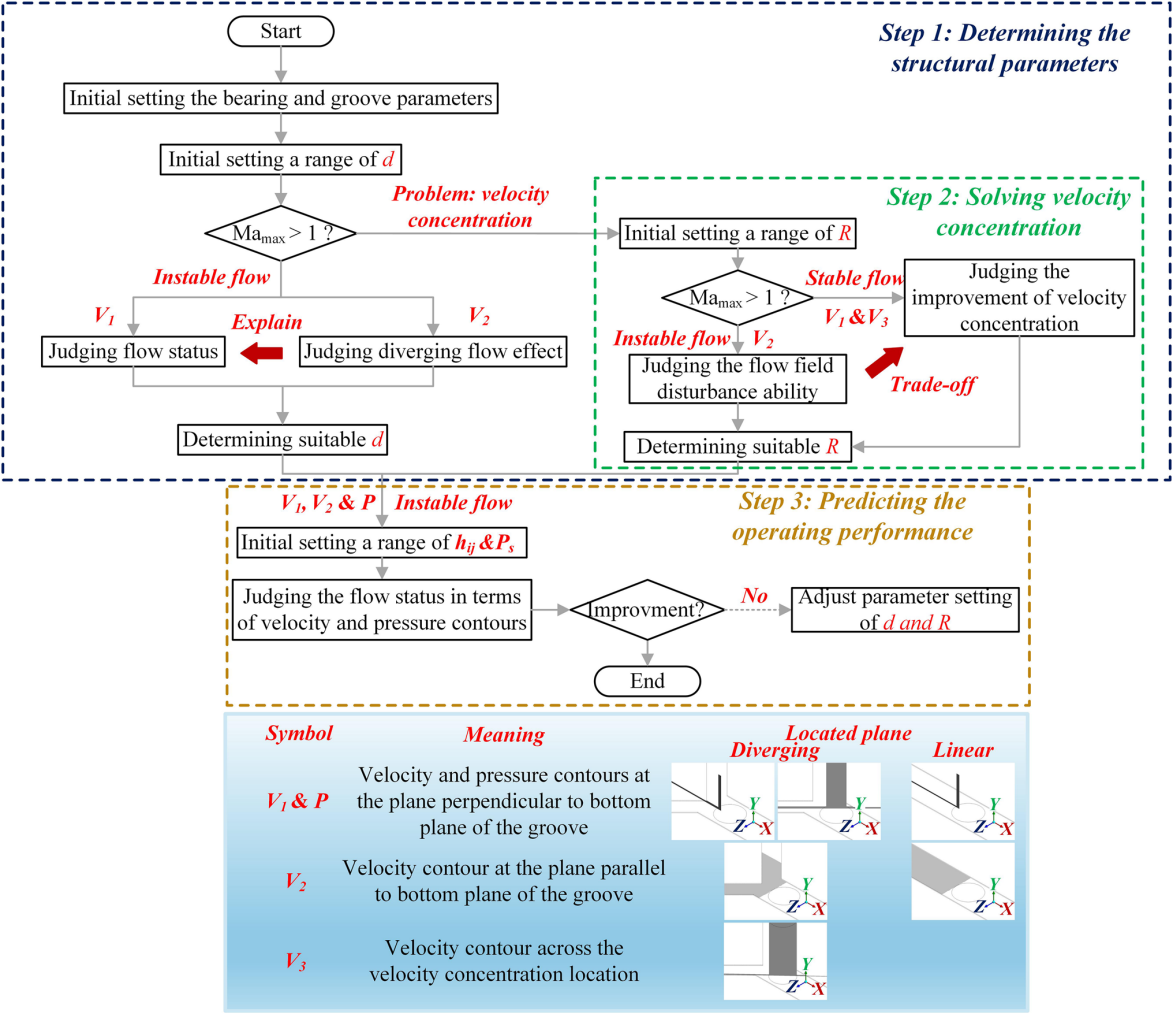
Numerical simulation flowchart

Figure 3 shows the design process flowchart of the proposed aerostatic restrictor based on CFD simulations. The symbols and the location plan of the CFD simulation results are defined in Fig. 3. Specifically, *V*_1_, *V*_2_, and *V*_3_ denote the airflow velocity distributions at cross-sections located sequentially from upstream to downstream of the bearing clearance, which are used to evaluate the evolution of supersonic flow and turbulence in the CFD analysis. The workflow mainly consists of three steps:

(i) Determination of *d* of the diverging groove restrictor: CFD simulations are used to investigate the influence of *d* on the airflow behavior in the grooves under unstable conditions, where Ma_max_ is over 1. Accordingly, suitable *d* can be determined.
(ii) Determination of *R* of the diverging groove restrictor: In (i), the velocity concentration, found in the diverging groove restrictor under stable flow, must be avoided by setting proper *R*. Simultaneously, the corresponding negative influence of the airflow status at unstable conditions should be considered.
(iii) Operating performance prediction: On the basis of the determined *d* and *R* in steps (i) and (ii), the operating performance of the proposed aerostatic restrictor is studied under different *h*_*ij*_ and *P*_s_ values, which guide the final determination of the restrictor key structural parameters.

Both stable and unstable flow characteristics of the proposed diverging groove restrictor, during structural parameter determination and operating performance prediction, were compared with those of conventional linear groove restrictors.

### CFD Simulation Model

**Fig. 4:**
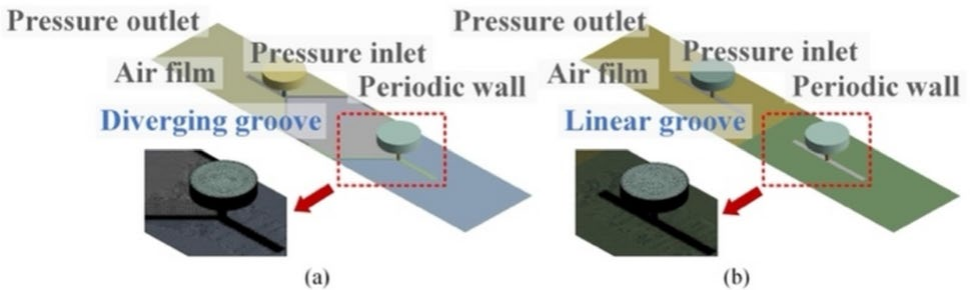
Boundary conditions and computational mesh for the **a** diverging and **b** linear groove restrictor models

To study the airflow characteristics in partial aerostatic bearings, three-dimensional numerical models of the diverging and linear groove restrictors were built using the ANSYS CFD FLUENT® software, as shown in Fig. 4. The geometric structure of each restrictor was simplified into an axially symmetric model with periodic boundary conditions to improve computational efficiency [8, 25]. Moreover, periodic boundaries were used to model repeating flow patterns efficiently while preserving physical accuracy. The inlet and outlet boundaries were assigned as constant absolute pressure and atmospheric pressure, respectively (Fig. 4a and b). All remaining solid surfaces were treated as stationary and impermeable walls. The cone angle for the air supply passages of both bearings was 120°. The bearing clearance was primarily meshed using structured hexahedral sweep elements with an element size of 0.002 mm. To enhance computational accuracy, local refinement with an element size of 0.01 mm was conducted at the diverging and linear grooves, highlighted by red boxes in Fig. 4.

The Reynolds number (*Re*) of the pressurized bearing clearance of both aerostatic bearings can be described as follows [26]:

1 $$Re=\frac{\rho v{d}_{\mathrm{s}}}{\mu }$$

where $\rho$, $v$, and $\mu$ represent the density, the maximum velocity, and the viscosity of the flow air, respectively. ${d}_{\mathrm{s}}$ represents the dimensional size parameter of the air film of the aerostatic bearing. The calculated *Re* of all the following simulation cases is less than 3000 under the set *h*_*ij*_ and *P*_s_, indicating the suitability of the laminar flow model [26–28]. Then, the steady flow calculation is set as it can reduce computational expense with satisfactory accuracy [29, 30].

To ensure the reliability of the simulation results, a mesh independence study was conducted before the main analysis tasks. The parameters used to evaluate mesh independence for both aerostatic restrictors are presented in Table 1. The carrying loads under varying mesh densities were selected as key indices for assessing the accuracy of the simulation results [30]. Meanwhile, Table 2 shows that the variation in carrying loads across different mesh configurations can be negligible when changing the setting conditions from 2 to 3. This indicated that the simulation results were not influenced by the mesh element setting. Thus, the built simulation models met accuracy requirements and were suitable for further analyses.

**Table 1:** Setting parameters for mesh independence evaluation

Parameters	Value
Absolute supply pressure *P*_s_	0.6 MPa
Atmosphere pressure* P*_a_	0.1 MPa
Film thickness *h*_*ij*_	20 µm
Orifice distance *d* (diverging)	4.7 mm
Round corner *R* (diverging)	0.2 mm

**Table 2:** Detailed information on meshes under various elements

Bearing types	Condition	No. of elements	Carrying load	Change rate
Diverging groove	1	3,035,211	82.245337	
	2	4,983,760	82.163174	0.1%
	3	7,996,226	82.097496	0.08%
Linear groove	1	4,832,109	91.034652	
	2	6,697,011	90.961883	0.08%
	3	8,985,032	90.898254	0.07%

## Analysis of CFD Simulation Results

### Effects of Key Structural Parameters on Airflow Status

**Fig. 5:**
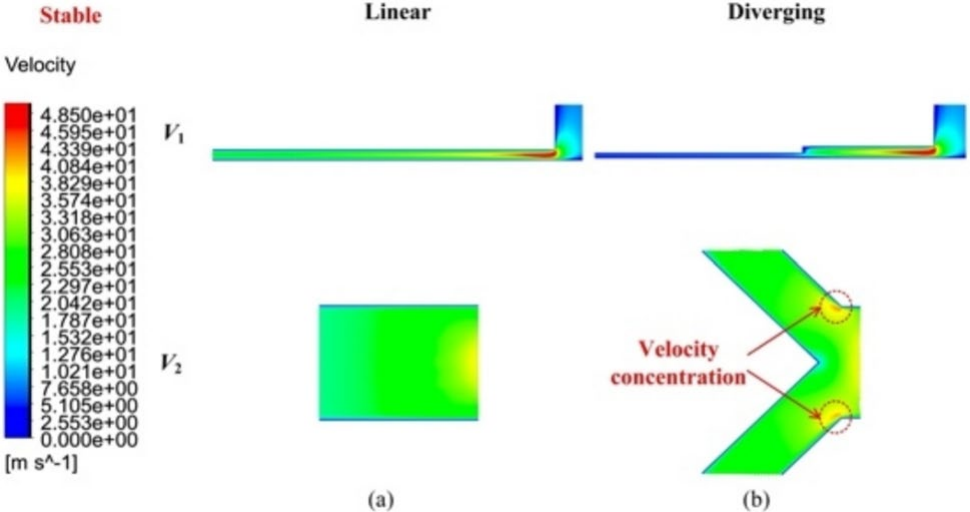
Velocity distribution contours *V*_1_ and *V*_2_ of the **a** linear and **b** diverging groove restrictors in air film under stable flow conditions

**Fig. 6:**
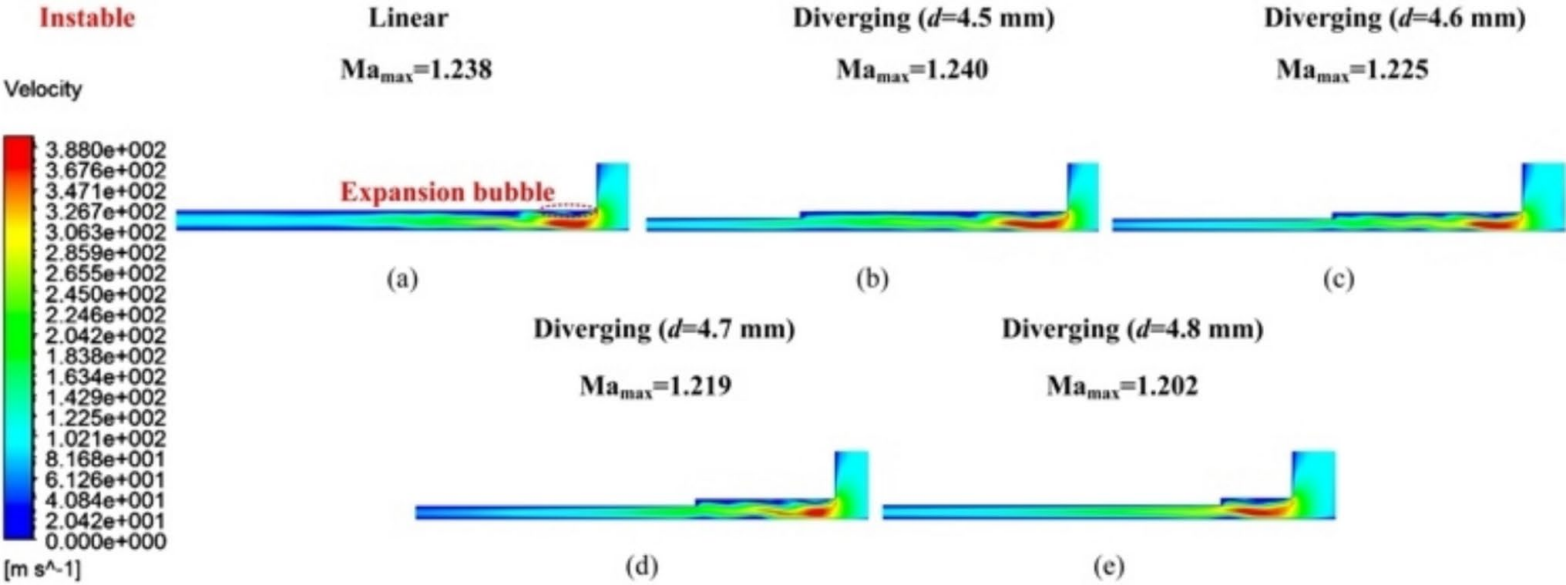
Velocity distribution contour *V*_1_ of **a** the linear groove restrictor, **b** *d* = 4.5 mm, **c** *d* = 4.6 mm, **d** *d* = 4.7 mm, and **e** *d* = 4.8 mm of the diverging groove restrictor under unstable flow conditions

**Fig. 7:**
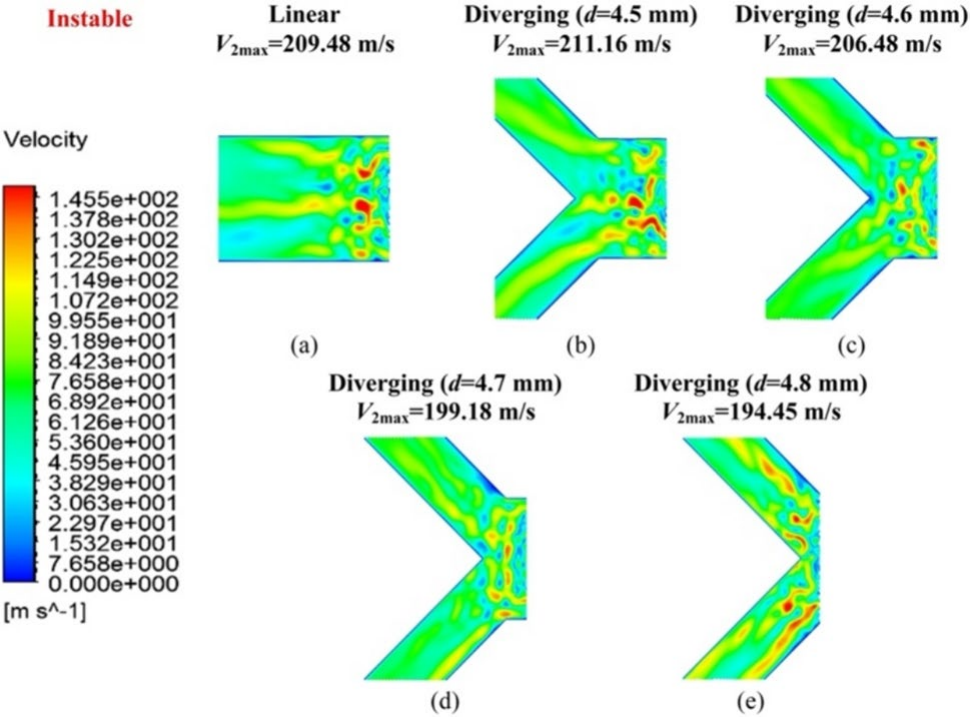
Velocity distribution contour *V*_2_ of **a** the linear groove restrictor, **b** *d* = 4.5 mm, **c** *d* = 4.6 mm, **d** *d* = 4.7 mm, and **e** *d* = 4.8 mm of the diverging groove restrictor under unstable flow conditions

Figures 5, 6, 7 show the velocity distribution contours* V*_1_ and *V*_2_ under the stable and unstable conditions inside the pressurized air film, respectively, corresponding to *h*_*ij*_ of 8 and 20 µm at a *P*_s_ value of 0.5 MPa.

Under stable flow, as shown in *V*_1_ of Fig. 5, the maximum air velocity was observed near the supply hole. As the maximum air velocities at *V*_1_ and *V*_2_ were significantly lower than the speed of sound, no evident sonic region was present in either the diverging or linear groove restrictors. Consequently, the airflow velocity tended to decrease evenly from the feed hole region toward the atmosphere. However, a localized region of velocity concentration formed in the diverging groove restrictor (Fig. 5b), which negatively affected the overall airflow behavior but could be mitigated by setting *R*.

Conversely, under unstable flow (Figs. 6 and 7), the maximum airflow velocity at *V*_1_ exceeded the speed of sound (Ma_max_ larger than 1), causing the formation of expansion bubbles in both restrictors, as marked in Fig. 6a. This phenomenon induced shock waves, which generated an adverse pressure gradient that promoted the development of sonic regions [26, 28]. Additionally, the airflow velocity distribution at *V*_2_ in Fig. 7 exhibited nonuniformity from upstream to downstream, suggesting the presence of turbulent flow. This unstable airflow status can severely impact the bearing dynamic stability.

When *d* = 4.5 or 4.6 mm, as shown in Fig. 7b and c, the proposed diverging groove restrictor exhibited a maximum velocity value in *V*_2_ (i.e., *V*_2max_) that was either larger than or comparable to that of the linear groove restrictor (Fig. 7a). This indicated that, at these dimensional parameters, the proposed restrictor did not evidently influence the airflow characteristics, likely because the airflow was diverged at the remote and downstream positions of the sonic region.

However, when *d* increased to 4.7 mm, the air velocity distribution in *V*_2_ of the diverging groove restrictor started to change. A slightly unstable airflow phenomenon began to emerge within the inclined grooves, as illustrated in Fig. 7d. Interestingly, this instability appeared to be positively mitigated by the geometry of the diverging groove, which contributed to a more controlled flow status. At this dimension in Fig. 6d, Ma_max_ and *V*_2max_ dropped slightly to 1.219 and 199.18 m/s, respectively, compared with 1.238 and 209.48 m/s for the linear groove restrictor. This indicates that the divergent inclined groove can modestly suppress instability in pressurized airflow.

When *d* was 4.8 mm, although *V*_2max_ in Fig. 7e further decreased to 194.45 m/s, an evident uneven velocity distribution was observed in *V*_2_, indicating that the unstable airflow was then being directed into both inclined grooves. In such a case, the turbulent airflow generated farther downstream from the inlet hole can lead to microvibrations in aerostatic bearings, which are detrimental to operational stability and must be avoided [14]. Thus,* d* = 4.7 mm was selected as the designed geometrical parameter for the proposed diverging groove restrictor.

**Fig. 8:**
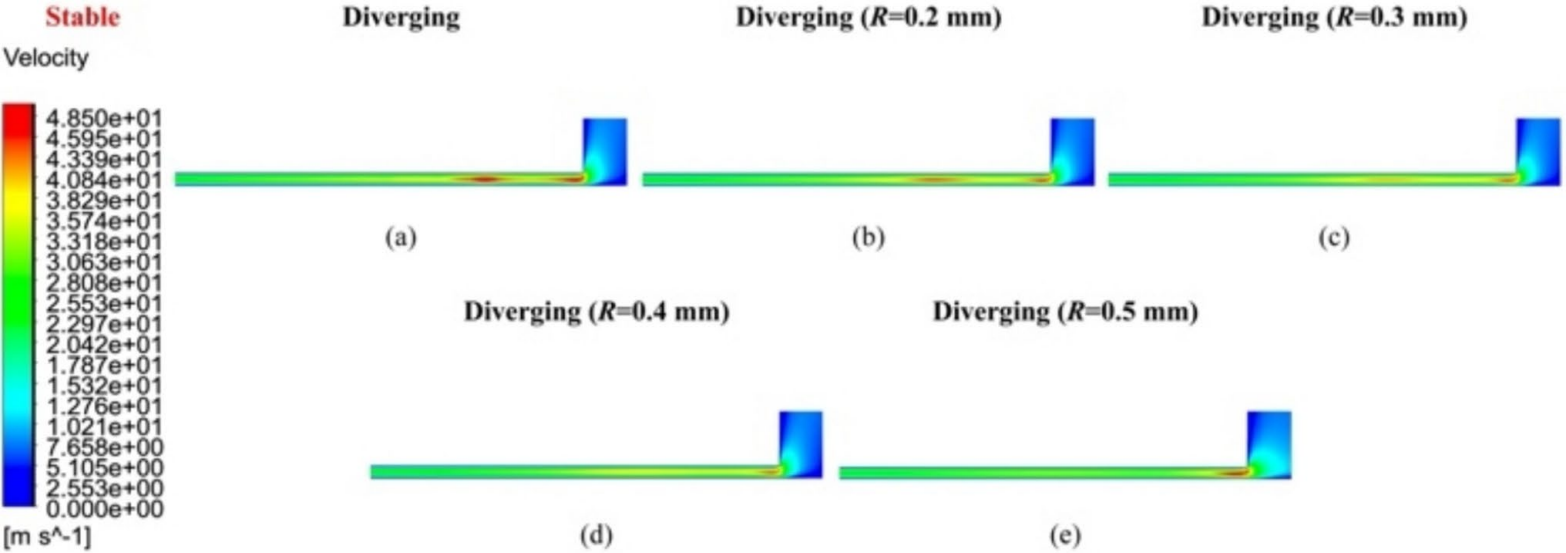
Velocity distribution contour *V*_3_ of **a** the linear groove restrictor, **b** *R* = 0.2 mm, **c** *R* = 0.3 mm, **d** *R* = 0.4 mm, and **e** *R* = 0.5 mm of the diverging groove restrictor under stable flow conditions

**Fig. 9:**
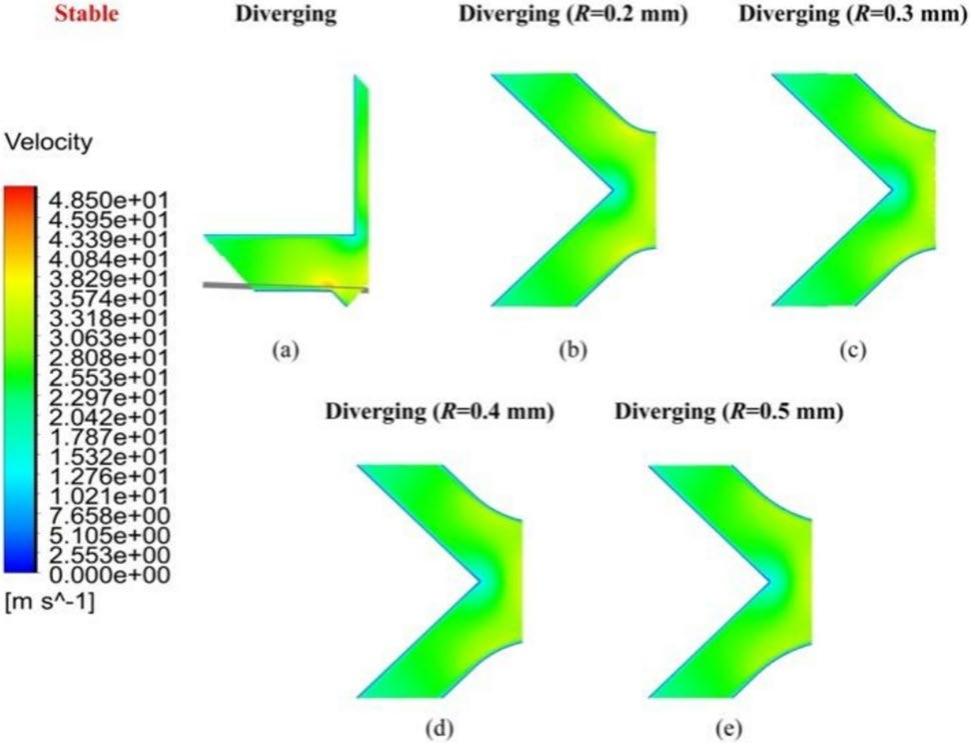
Velocity distribution contour *V*_2_ of **a** *R* = 0 mm, **b** *R* = 0.2 mm, **c** *R* = 0.3 mm, **d** *R* = 0.4 mm, and **e** *R* = 0.5 mm of the diverging groove restrictor under stable flow conditions

Figures 8 and 9 compare the velocity distribution contours of *V*_3_ and *V*_2_ under stable airflow, influenced by the geometrical parameter *R*. Specifically, as shown in *V*_2_ of Fig. 9a, although the flow velocity decreased uniformly from upstream to downstream (relative to the feedhole position), a notable velocity concentration was observed in the diverging groove restrictor without a rounding corner. The airflow velocity distribution in *V*_3_ of Fig. 8a also demonstrated two distinct regions with high velocity values. As *R* increased from 0.2 to 0.5 mm in Fig. 9b–e, both intensity and extent of the velocity concentration area gradually diminished, ultimately disappearing when *R* reached 0.5 mm.

**Fig. 10:**
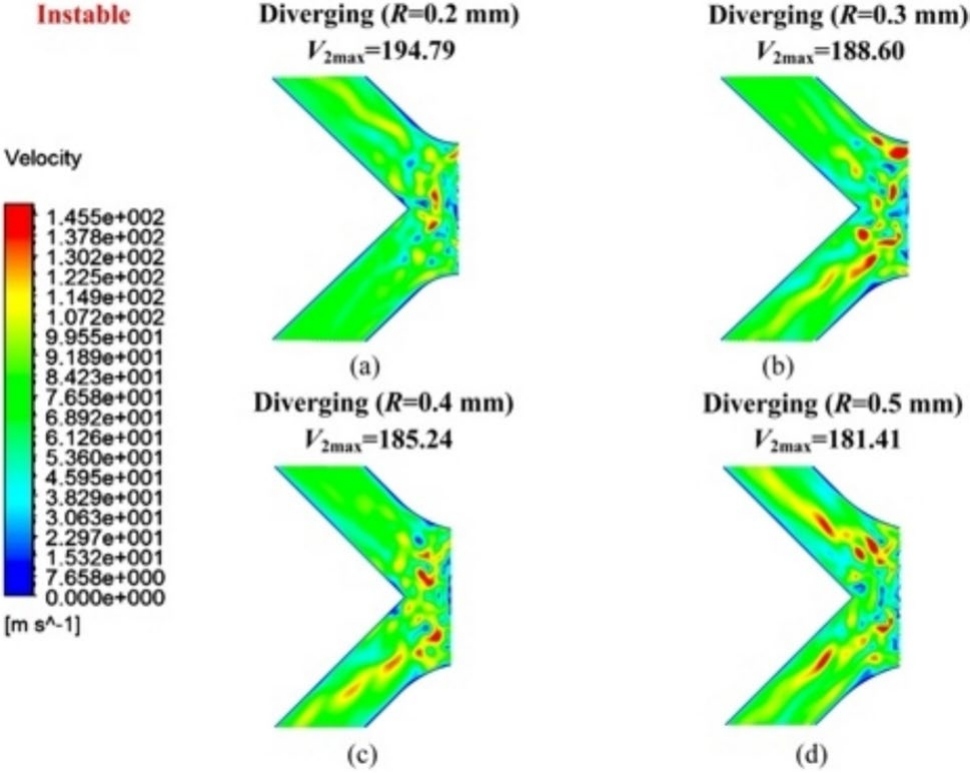
Velocity distribution contour *V*_2_ of **a** *R* = 0.2 mm, **b** *R* = 0.3 mm, **c** *R* = 0.4 mm, and **d** *R* = 0.5 mm of the diverging groove under unstable flow conditions

Figure 10 shows the velocity distribution contour of the diverging groove restrictor under unstable flow. When *R* was set to 0.5 mm in Fig. 10d, although *V*_2max_ was minimized at 181.41 m/s and the velocity concentration was reduced, unstable airflow was still easily directed into the inclined grooves. This indicated that reducing velocity concentration alone does not guarantee the effective suppression of unstable airflow.

A similar phenomenon was observed when *R* changed to 0.4 and 0.3 mm in Fig. 10c and b, with slightly increased *V*_2max_ but with the continued presence of airflow instability. However, when *R* was further decreased to 0.2 mm in Fig. 10a, *V*_2max_ reached a maximum value of 194.79 m/s, and no evident unstable airflow appeared within the inclined grooves. This suggests that a smaller *R*, despite the existing velocity concentration, can more effectively prevent unstable airflow from entering inclined grooves. Thus, considering the velocity distributions under both stable and unstable flow conditions, *R* = 0.2 mm was finally determined to provide a balance between velocity concentration control and air flow stability.

The values *d* = 4.7 mm and *R* = 0.2 mm were selected because the corner radius suppressed velocity concentration under stable flow, whereas the orifice distance reduced the Mach number and turbulence under unstable supersonic flow.

### Flow Details under Different Operating Conditions

Figures 5, 6, 7 show the velocity distribution contours* V*_1_ and *V*_2_ under stable and unstable flow inside the pressurized air film, corresponding to *h*_*ij*_ of 8 and 20 µm at *P*_s_ of 0.5 MPa. To verify the effectiveness of the proposed design in enhancing the dynamic stability of aerostatic bearings, CFD simulations were conducted to investigate the airflow status under varying *h*_*ij*_ and *P*_s_ conditions. According to the bearing operating principle, a higher *h*_*ij*_ typically requires a larger mass flow rate to fulfill the bearing clearance. Consequently, the speed of the supply pressurized air must be significantly increased, which inevitably alters airflow behaviors.

**Fig. 11:**
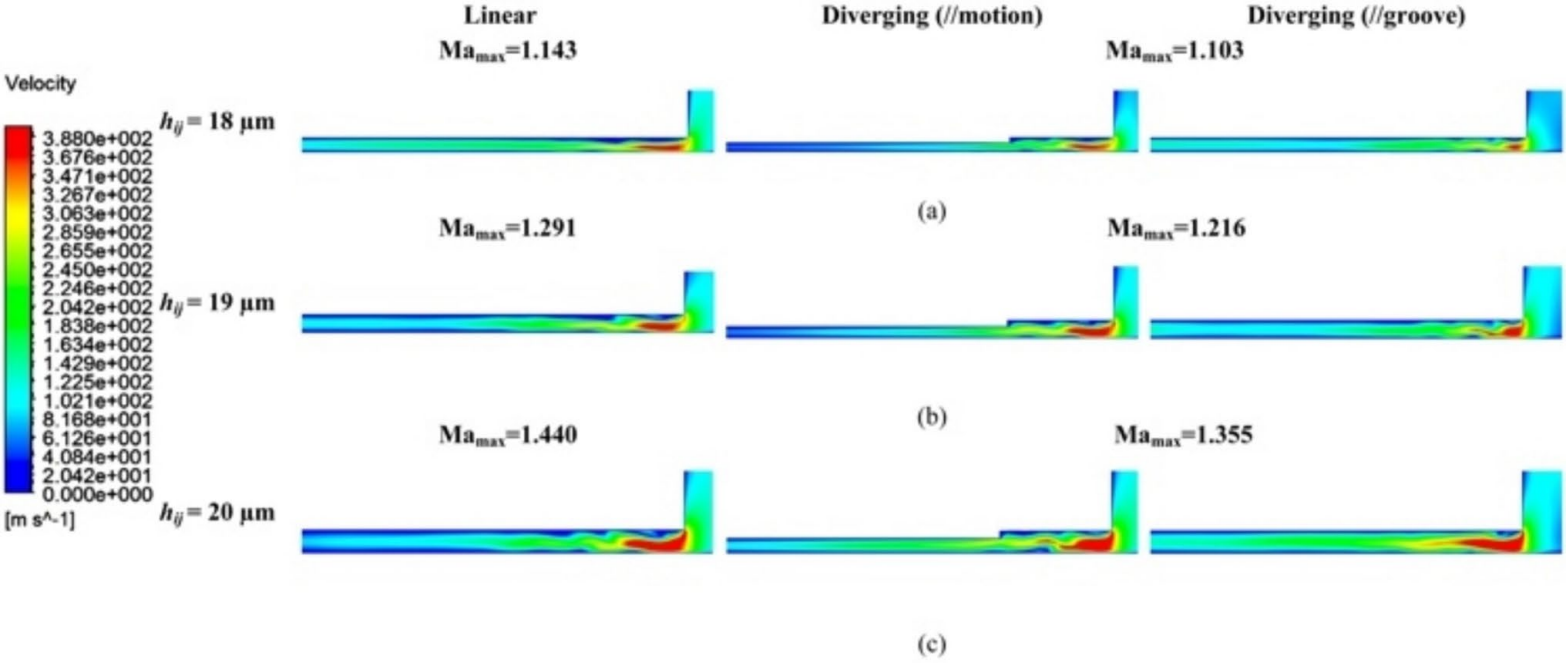
Velocity distribution contour *V*_1_ under different *h*_*ij*_ of **a** 18 µm, **b** 19 µm, and **c** 20 µm of the linear and diverging groove restrictors

For both linear and diverging groove restrictors, Ma_max_ of the maximum airflow velocity at *V*_1_ was obviously larger than 1 in Fig. 11, suggesting that turbulent flow dominated the pressurized airflow status. When *h*_*ij*_ ranged from 18 to 20 μm, this turbulent behavior likely induced significant microvibrations. As *h*_*ij*_ increased, the instability of aerostatic bearings intensified, as evidenced by an increase in Ma_max_ from 1.143 to 1.440 for the linear groove restrictor and 1.103 to 1.355 for the diverging groove restrictor. This phenomenon was primarily attributed to the increased mass flow rate of the supplied pressurized air at larger *h*_*ij*_ values. Notably, the diverging groove restrictor showed lower Ma_max_ values in all cases compared with the linear groove restrictor. This demonstrates the superior dynamic stability of the proposed design, making it a suitable candidate for high-precision applications.

**Fig. 12:**
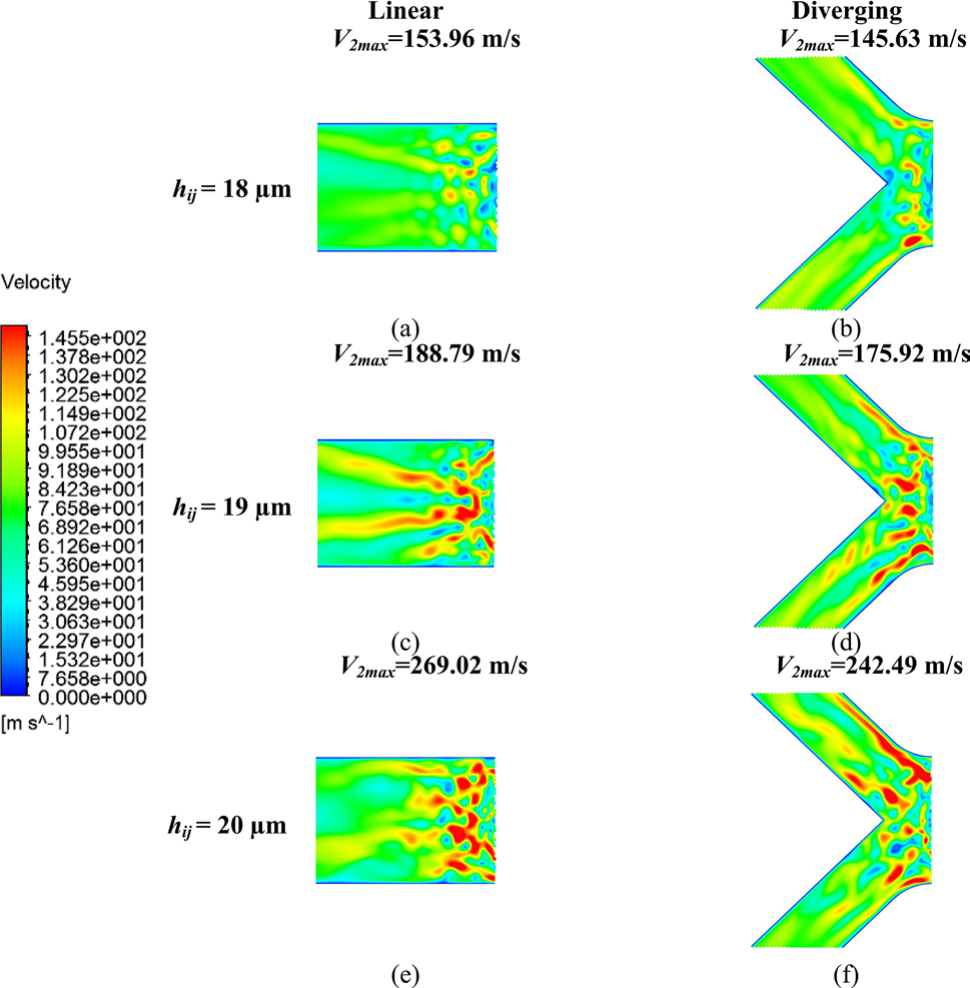
Velocity distribution contour *V*_2_ of **a** 18 µm, **c** 19 µm, and **e** 20 µm of the linear groove restrictor; and **b** 18 µm, **d** 19 µm, and **f** 20 µm of the diverging groove restrictor

The velocity distribution in *V*_2_, as shown in Fig. 12, revealed that airflow in both linear and diverging groove restrictors became increasingly unstable as *h*_*ij*_ increased. This instability was further demonstrated by the rise in *V*_2max_ from 159.96 to 269.02 m/s in the linear groove restrictor and from 145.63 to 242.49 m/s in the diverging groove restrictor. Moreover, the comparatively lower *V*_2max_ in the diverging groove restrictor also indicated that it maintained better dynamic stability under the same conditions.

As more pressurized airflow entered the expanded bearing clearance, a greater volume of unstable airflow was forced into the inclined grooves of the diverging groove restrictor, intensifying turbulence and then compromising the bearing stability (Fig. 12b, d, and f). Moreover, it was reinforced by the expansion of the high-speed airflow region parallel to the inclined groove in *V*_1_ in Fig. 11 as *h*_*ij*_ increased.

Overall, these comparisons confirm that the diverging groove restrictor achieved lower Ma_max_ and suppressed turbulence intensity compared with the linear groove restrictor under supersonic airflow conditions, thereby validating the proposed design as an effective approach for enhancing bearing dynamic stability.

**Fig. 13:**
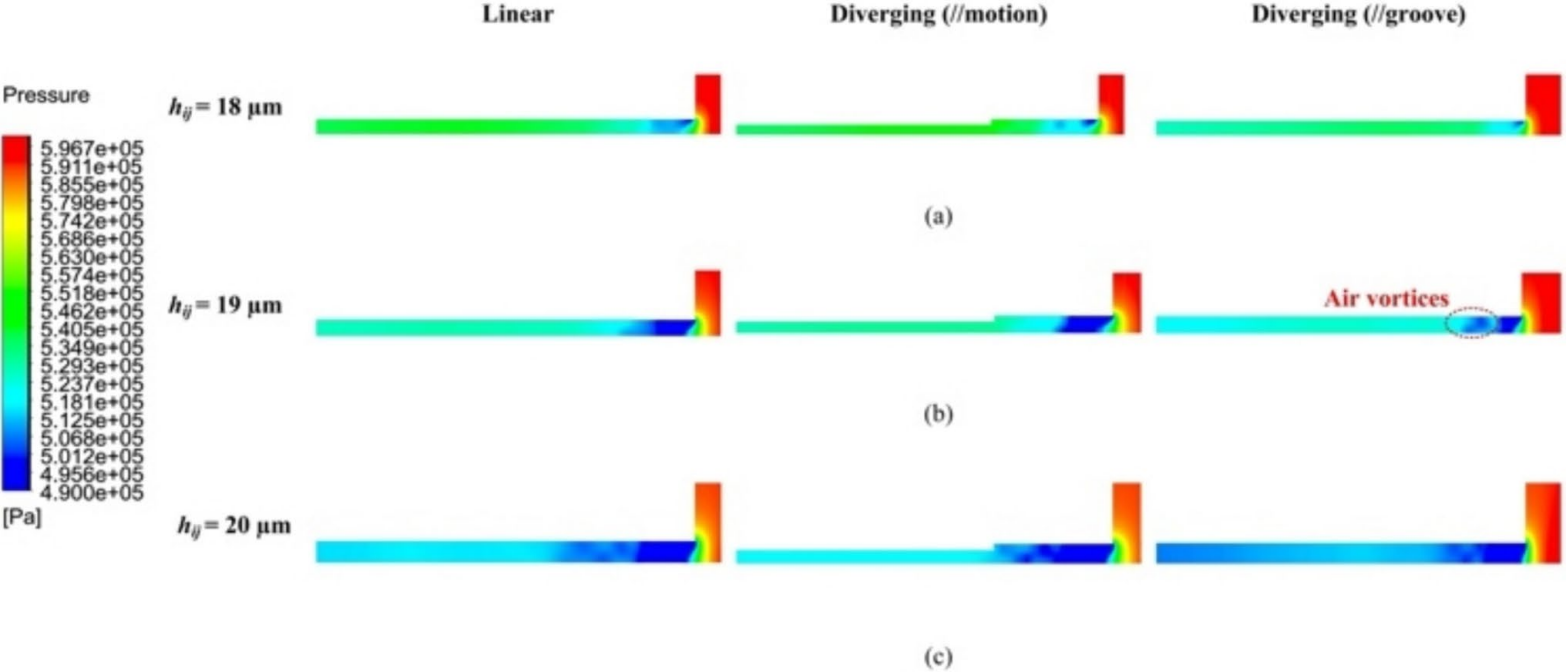
Pressure distribution contour *P* under different *h*_i__j_ of **a** 18 µm, **b** 19 µm, and **c** 20 µm of the linear and diverging groove restrictors

Figure 13 shows the pressure distribution contour *P* of both linear and diverging groove restrictors under different *h*_*ij*_ values. A pronounced pressure depression near the orifice inlet was observed, resulting from the supersonic pressurized airflow abruptly turning 90° upon encountering the fixed wall side. As *h*_*ij*_ increased from 18 to 20 μm, the pressure depression area obviously expanded, as shown in Fig. 13a and b for both restrictor types. Downstream of the pressure depression area, turbulence arose in the form of air vortices, as marked in Fig. 13b. The airflow vortex center, located at the minimum pressure points, led to noticeable variations in the local pressure distribution field [31]. When *h*_*ij*_ reached 20 μm in Fig. 13c, more vortices formed compared with those at 19 μm in Fig. 13b, indicating heightened airflow instability. Compared with the linear groove restrictor, the proposed diverging groove restrictor demonstrated vortices originating closer to the pressure depression area, implying the better microvibration suppression of the proposed aerostatic bearing.

**Fig. 14:**
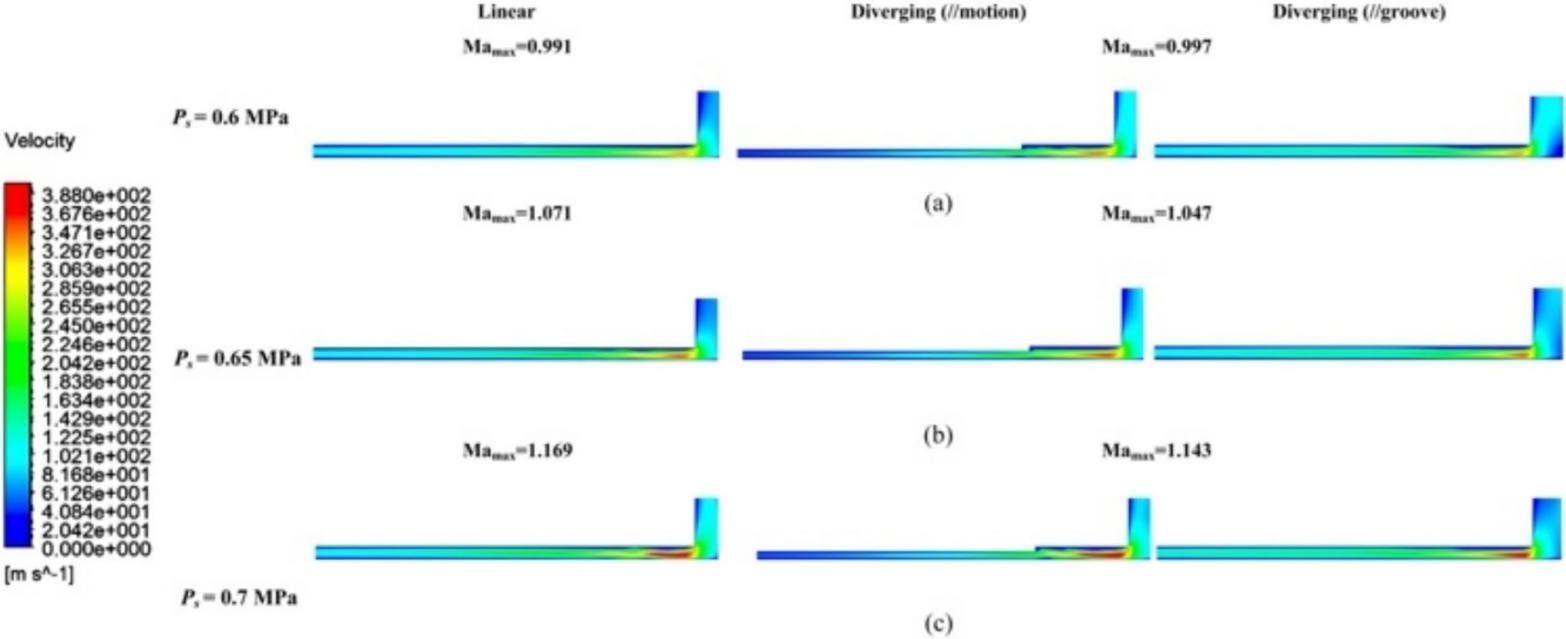
Velocity distribution contour *V*_1_ under different *P*_s_ of **a** 0.6 MPa, **b** 0.65 MPa, and **c** 0.7 MPa of the linear and diverging groove restrictors

To further investigate the dynamic behavior of the pressurized airflow, the influence of the input *P*_s_ on pressurized airflow status is explored herein, in contrast to the instability driven by variations in *h*_*ij*_ described in Sect. 4.2. Under different *P*_s_ values, Ma_max_ at *V*_1_ in Fig. 14b and c slightly exceeded 1, indicating the onset of local supersonic airflow. Specifically, at 0.65 and 0.7 MPa, the Ma_max_ values were 1.071 and 1.169 for the linear groove restrictor, respectively, whereas they reached 1.047 and 1.143 for the diverging groove restrictor. However, at 0.6 MPa in Fig. 14a, both restrictors exhibited values below 1, indicating the existence of a subsonic and stable airflow. This could be explained by the limited bearing clearance of 17 μm restricting volumetric airflow, preventing the attainment of supersonic speeds—unlike the larger clearances analyzed in Sect. 4.2.

The corresponding airflow dynamic mechanism can be explained using the Laval nozzle principle. In subsonic fluid velocity conditions, the narrowing geometry of the diverging groove generates an adverse pressure gradient, consequently accelerating the airflow velocity. This is reflected by the observed Ma_max_ values, with the linear groove restrictor having a lower Ma_max_ of 0.991 compared with 0.997 for the diverging groove restrictor (Fig. 14a). However, at the supersonic fluid status depicted in Fig. 14b and c, the diverging groove structure exerts a decelerating effect on the airflow velocity relative to the linear groove structure, directly contributing to improving the dynamic stability of the aerostatic bearing.

**Fig. 15:**
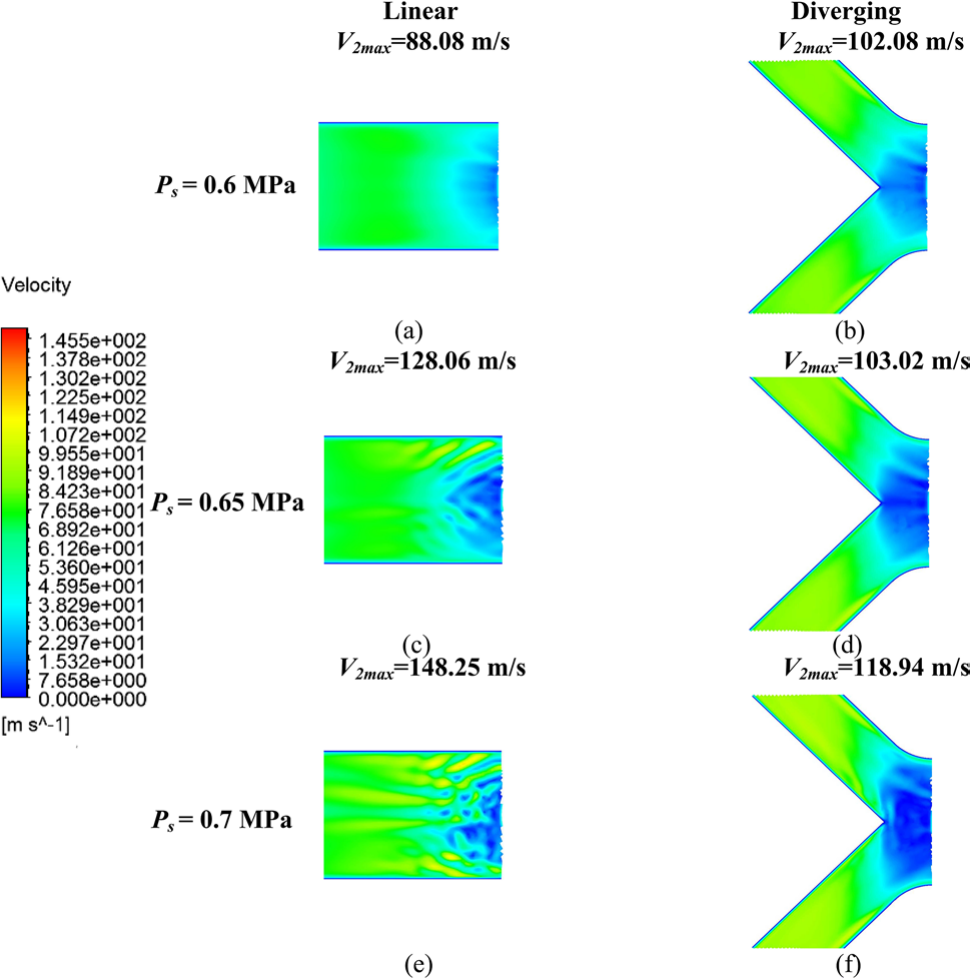
Velocity distribution contour *V*_2_ of **a** 0.6 MPa, **c** 0.65 MPa, and **e** 0.7 MPa of the linear groove restrictor; and **b** 0.6 MPa, **d** 0.65 MPa, and **f** 0.7 MPa of the diverging groove restrictor

Figure 15 presents the airflow velocity distribution in *V*_2_ for both restrictors. As shown in Fig. 15a, at a *P*_s_ of 0.6 MPa, *V*_2max_ within the linear groove restrictor was only 88.08 m/s, whereas it increased to 102.08 m/s for the proposed diverging groove restrictor in Fig. 15b. This difference was due to the larger low-velocity region of the diverging groove restrictor in Fig. 15b, which also indicated the formation of an expansion bubble. This bubble facilitated the increase in the airflow velocity through the adverse pressure gradient generated by shock waves. Then, as *P*_s_ rose, the expansion bubble continued to develop in Fig. 15c and e for the linear groove restrictor and in Fig. 15d and f for the diverging groove restrictor, leading to a higher Ma_max_, as shown in Fig. 14b and c.

As *P*_s_ reached 0.65 and 0.7 MPa, *V*_2max_ in the linear groove restrictor increased to 128.06 and 148.25 m/s in Fig. 15c and e, respectively, whereas those in the diverging groove restrictor were only 103.02 and 118.94 m/s in Fig. 15d and f. These findings highlight the role of the proposed diverging groove structures in reducing supersonic air velocity and suppressing unstable flow. The obvious turbulent flows inside the linear groove occurred behind the low-velocity region in Fig. 15c and e, which contrasted sharply with the almost stable flow inside the inclined grooves of the diverging groove restrictor in Fig. 15d and f. These results confirm the effectiveness of the diverging groove structure in controlling and stabilizing turbulent airflow.

**Fig. 16:**
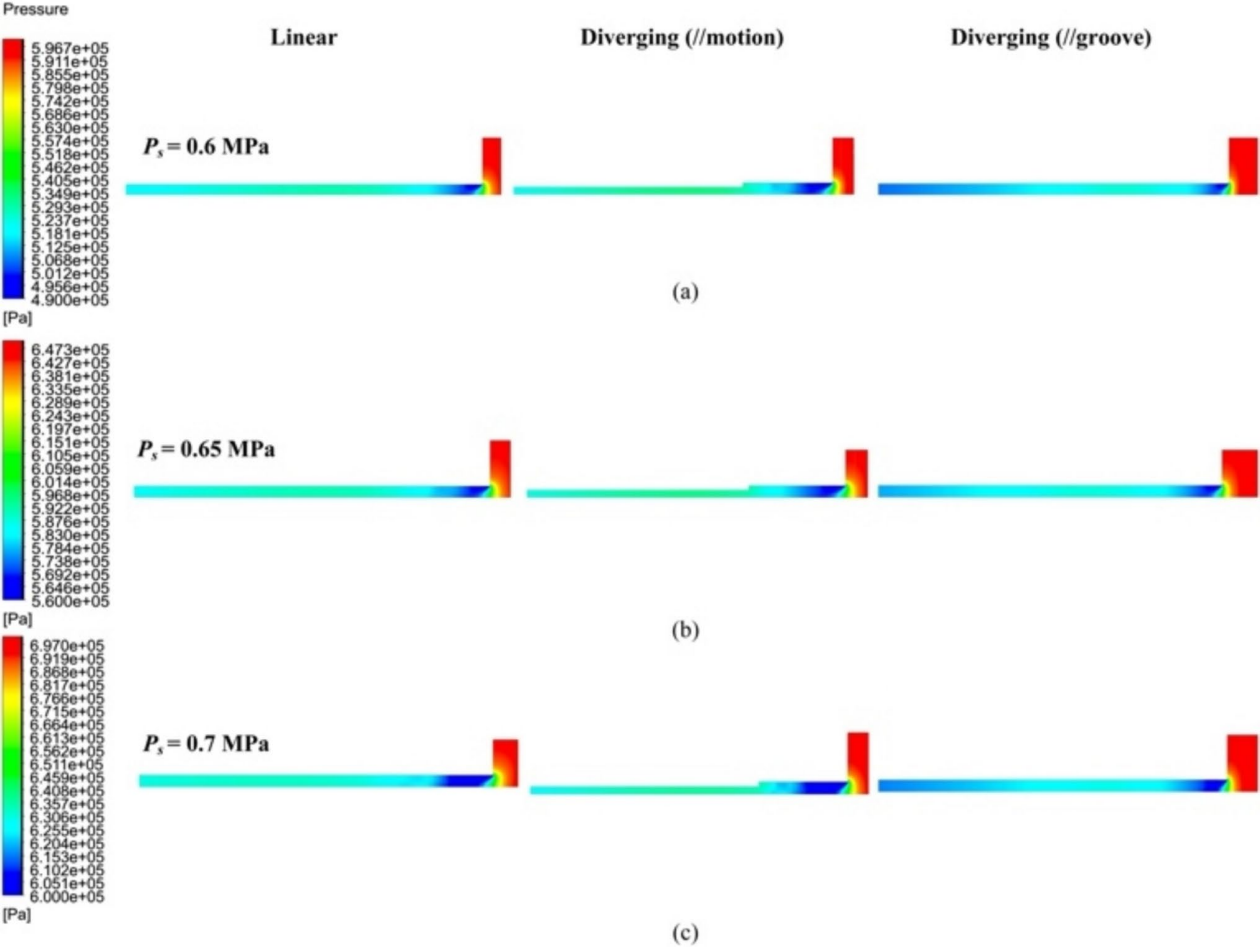
Pressure distribution contour *P* under different *P*_s_ of **a** 0.6 MPa, **b** 0.65 MPa, and **c** 0.7 MPa of the linear and diverging groove restrictors

The pressure distributions of the linear and diverging groove restrictors under different *P*_s_ values are illustrated in Fig. 16. At a *P*_s_ of 0.6 MPa in Fig. 16a, an obvious expanded pressure depression area accompanied by the formation of air vortices could be observed in the diverging groove restrictor, although no obvious turbulent flow entered into the inclined grooves. In contrast, the linear groove restrictor showed a smaller pressure depression area.

As *P*_s_ increased to 0.65 and 0.7 MPa in Fig. 16b and c, the pressure depression area of both restrictors expanded. However, the proposed diverging groove restrictor performed better in terms of turbulent flow control. Specifically, more air vortices were found downstream of the pressure depression area inside the linear groove compared with those within the diverging groove.

Overall, the above CFD simulations indicate that the proposed Laval-nozzle-inspired diverging groove structures can reduce or minimize the negative influence of airflow with supersonic speed, thereby mitigating or eliminating the formation of unstable airflow (i.e., turbulence). Consequently, the dynamic stability of aerostatic bearings can be improved.

## Experimental Study on the Dynamic Stability of Aerostatic Bearings

### Bearing Testing Setup

**Fig. 17:**
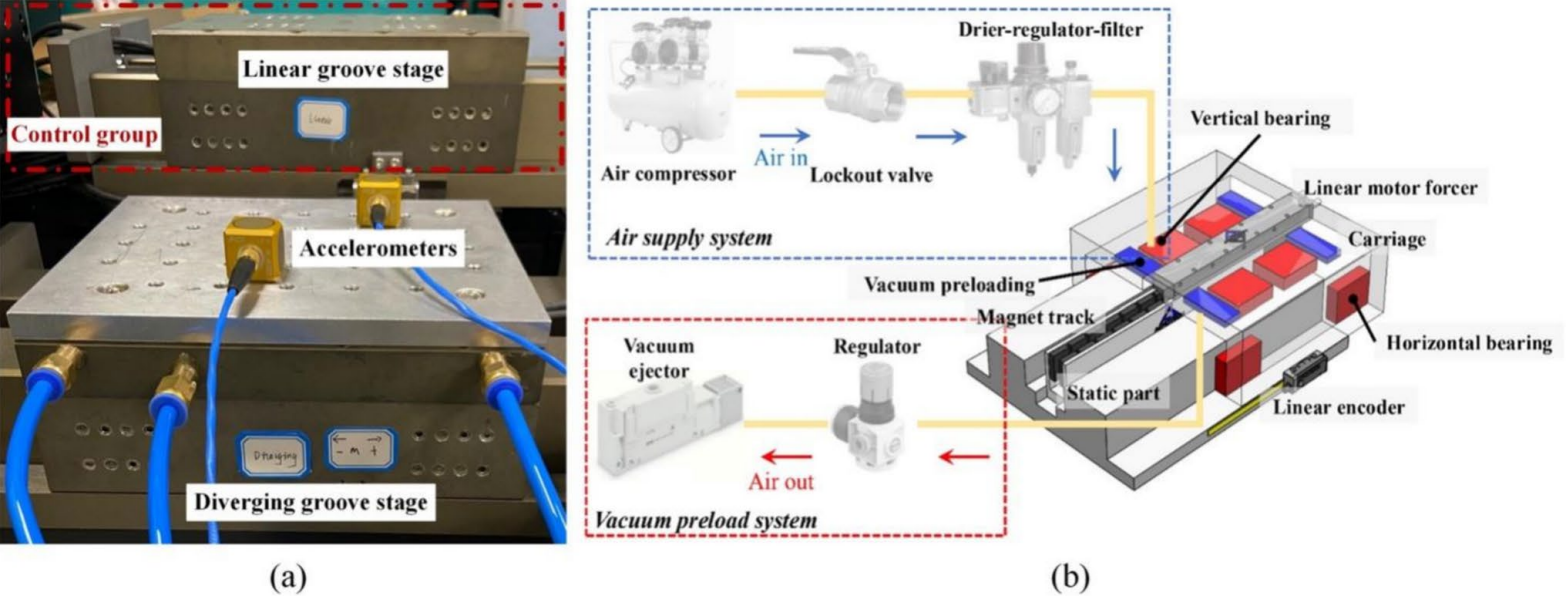
Testing experiment setting: **a** experimental setup for microvibration testing and **b** air supply and vacuum preloading systems

The dynamic stability of the aerostatic bearings equipped with the designed diverging and linear groove restrictors was evaluated and compared using a linear motion stage, as shown in Fig. 17a. Both aerostatic bearings were employed to support the stage’s moving parts along the vertical direction, as shown in Fig. 17b. In microvibration testing, acceleration sensors (356B, PCB) were attached to the stage’s top surface to collect data. Data processing was conducted using a data acquisition card (USB-1608HS, Measurement Computing). The vibration data were tested three times to check repeatability and were averaged to minimize prediction errors.

Figure 17b also shows the air supply and preloading system. The air was pressurized by a compressor and then passed through a dryer, pressure gauge, and filter. To maintain the targeted *h*_*ij*_ under a specific *P*_s_, the attraction force was generated by the preloading system, which suctioned the gas mass flow using a vacuum ejector (ZL112A, SMC). The bearing clearance was measured using capacitance sensors (CP8.0–2.0–2.0, IBS) mounted through fixtures on the carriage against the static surface of the slide.

### Experimental Results

**Fig. 18:**
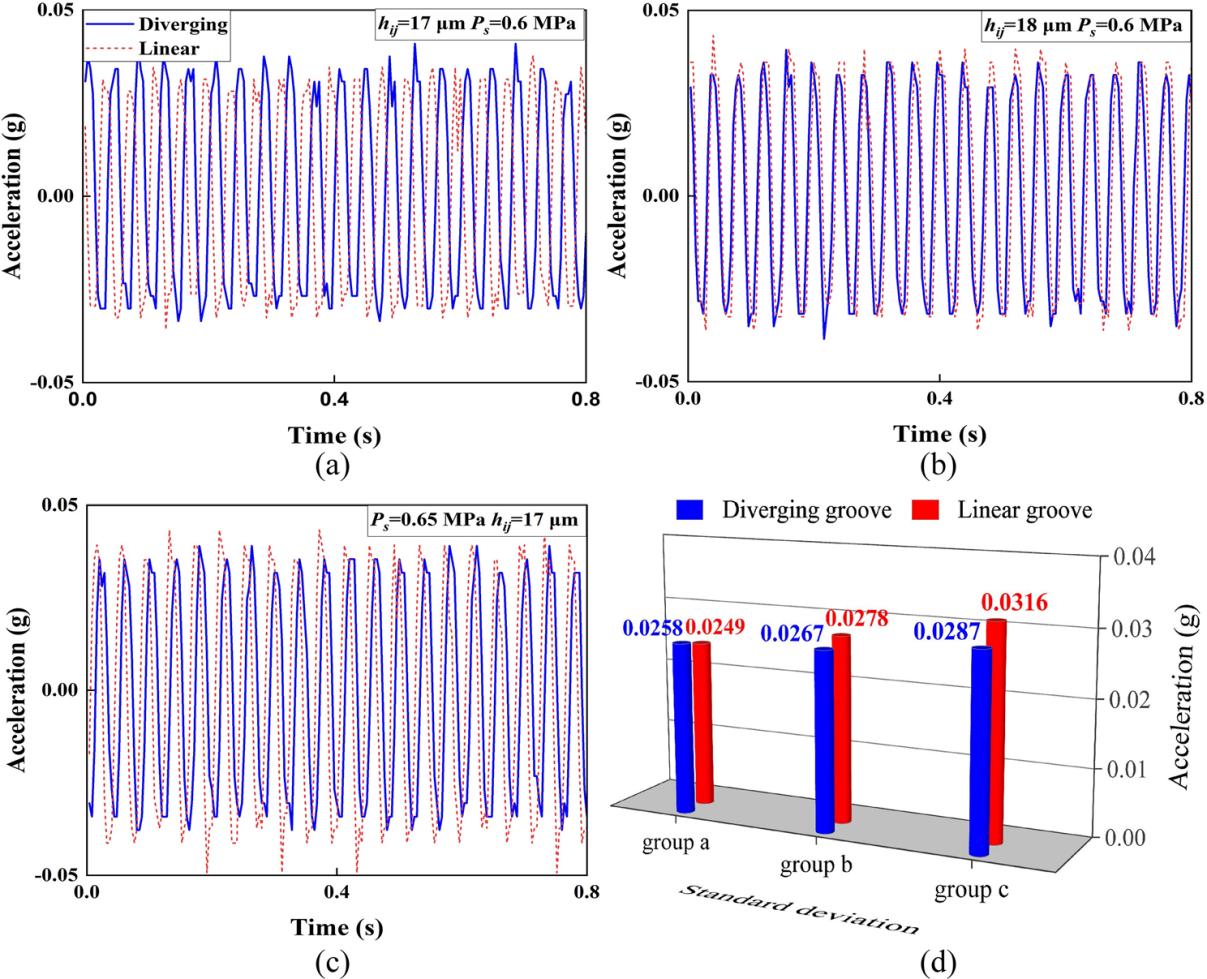
Vibration accelerations of the linear stage supported by the aerostatic bearings in the time domain under **a** group a: *h*_*ij*_ = 17 µm and *P*_s_ = 0.6 MPa; **b** group b: *h*_*ij*_ = 18 µm and *P*_s_ = 0.6 MPa; **c** group c: *P*_s_ = 0.65 MPa and *h*_*ij*_ = 17 µm; and **d** their SD comparison

To assess the dynamic performance of different bearing restrictor designs, Fig. 18 compares the vibration accelerations of the linear stage equipped with either aerostatic bearings with diverging or linear groove outlets. Measurements were conducted under varying *h*_*ij*_ (17–18 μm) and *P*_s_ (0.6–0.65 MPa). These conditions aligned with the parameter settings used in the CFD simulations discussed in Sect. 4.2.

As shown in Fig. 18, the diverging groove stage exhibited lower standard deviation (SD) values in the time domain (Fig. 18d) than the linear groove under supersonic conditions (0.0267 vs. 0.0278 in Fig. 18b and 0.0287 vs 0.0316 in Fig. 18c), confirming its suppression of microvibrations. Meanwhile, at subsonic conditions, the SD was slightly higher (0.0258 vs 0.0249 in Fig. 18a), which could be attributed to the negative influence of the Laval nozzle effect on airflow status at subsonic velocities, consistent with the simulation results in Figs. 14a and 15a.

**Fig. 19:**
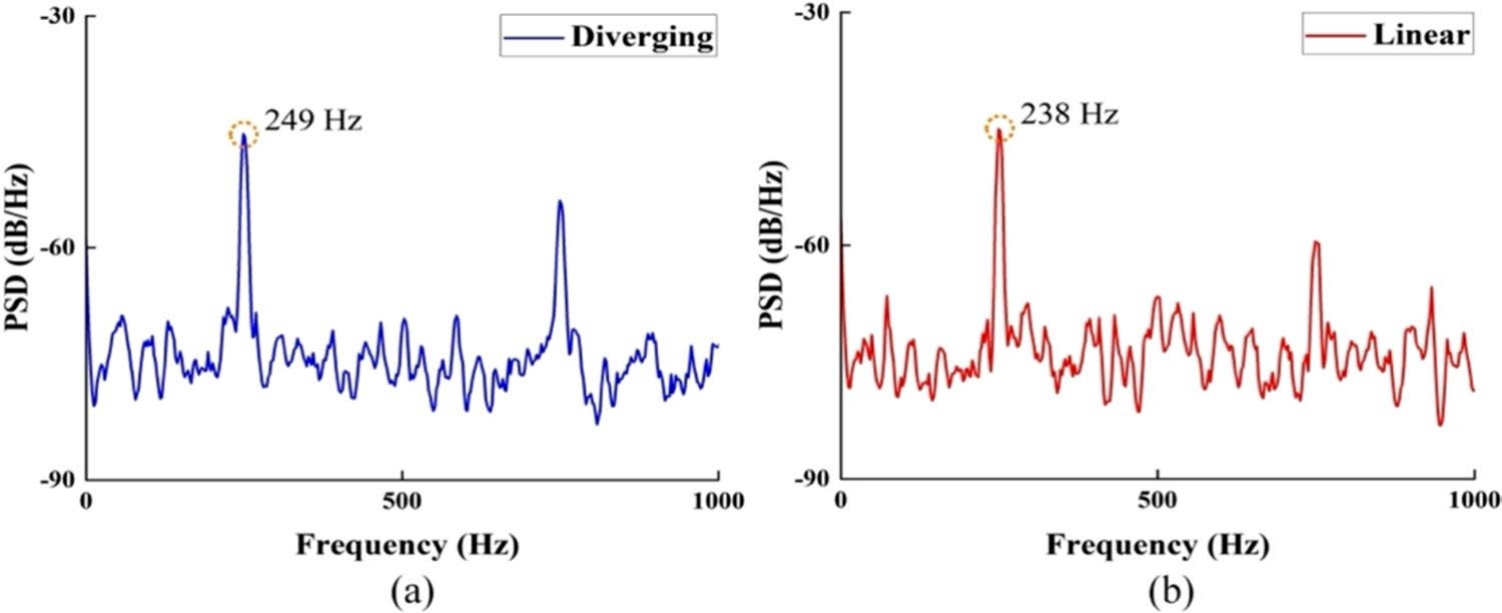
PSD of the **a** diverging and **b** linear groove stages at *h*_*ij*_ = 18 µm and *P*_s_ = 0.6 MPa, used to identify the resonant frequency

The power spectral density represents the distribution of vibration energy over frequency, enabling clear identification of dominant resonant modes relevant to microvibration behavior. As shown in Fig. 19, the dominant peaks at ~ 249 Hz for the diverging groove stage (Fig. 19a) and 238 Hz for the linear groove stage (Fig. 19b) corresponded to the primary natural frequency of the stage–bearing system. Importantly, the vibration amplitudes in Fig. 18 across all frequencies corresponded to nanometer-scale displacements, with maximum levels below 10 nm, confirming that the system operated within the expected microvibration range.

## Conclusions

In this work, the Laval nozzle principle was effectively incorporated into the design of an aerostatic bearing restrictor, presented in the form of diverging groove structures, aimed at decreasing supersonic flow velocity for resisting microvibrations. Using CFD simulations, this study revealed the influence of the selected structural parameters on the airflow status of the proposed diverging groove restrictor, which was compared with that of a linear groove restrictor. Then, key structural parameters were determined, and the operating performances were predicted. The effectiveness of the proposed aerostatic restrictor design in improving the dynamic stability of aerostatic bearings was verified experimentally. The main conclusions can be summarized as follows:

1. The study introduced a Laval-nozzle-inspired design for the outlet groove of an aerostatic restrictor. This geometrical design enhanced bearing dynamic stability by mitigating adverse pressure gradients by decreasing supersonic airflow velocity. To maximize pressurized airflow status control within the bearing clearance, the proposed diverging groove was designed with specific structural parameters—*d* = 4.7 mm and *R* = 0.2 mm—to reduce the negative diverging effect and the velocity concentration under unstable and stable flow, respectively.
2. This study investigated the underlying microvibration suppression mechanism using the designed aerostatic restrictor under operating *P*_s_ and *h*_*ij*_. Ma_max_ was determined to be strongly dependent on the air volume within the bearing clearance. Simulation results indicated that the proposed diverging groove restrictor achieved better dynamic stability compared with the conventional linear groove restrictor under supersonic conditions. However, it performed less effectively under subsonic conditions. These simulated results prove the existence of the Laval nozzle effect in the proposed diverging groove aerostatic restrictor.
3. Experimental tests validated the simulation results, confirming that the Laval nozzle principle was successfully applied to the aerostatic bearing stage prototype to suppress microvibrations under supersonic conditions. The tests further demonstrated that it may unintentionally increase subsonic airflow velocity because of the varying mechanisms of adverse pressure gradient regulation.

## Data Availability

The data underpinning this publication is available from the University of Strathclyde Knowledge Base.
